# Lost in Translation – eine Untersuchung der Höranstrengung und Performanz von Patient*innen mit Cochleaimplantat im erst- und fremdsprachlichen Setting

**DOI:** 10.1007/s00106-025-01666-5

**Published:** 2025-09-05

**Authors:** Susann Thyson, Simone Volpert, Maika Werminghaus, Laurenz Althaus, Thomas Klenzner

**Affiliations:** Hörzentrum Düsseldorf, Klinik für Hals-Nasen-Ohrenheilkunde, Medizinische Fakultät und Universitätsklinikum Düsseldorf, Heinrich-Heine-Universität Düsseldorf, Moorenstr. 5, 40225 Düsseldorf, Deutschland

**Keywords:** Mehrsprachigkeit, Rehabilitation, Sprachverstehen, Störschall, Migrationshintergrund, Multilingualism, Rehabilitation, Language comprehension, Speech in noise, Human migration

## Abstract

**Hintergrund und Ziel:**

Das Sprachverstehen in einer Fremdsprache stellt im Störschall eine erhöhte Anforderung dar. Für mehrsprachige Patient*innen mit Cochleaimplantat (PmCI) ergibt sich daraus eine besondere Herausforderung, da die audiometrische Routinediagnostik meist in der Umgebungssprache und nicht in der Erstsprache der Patient*innen erfolgt. Diese Studie untersucht deshalb das Sprachverstehen im Störschall sowie das subjektive Anstrengungsempfinden von PmCI im Vergleich zu normalhörenden Personen unter erst- und fremdsprachlichen Bedingungen.

**Material und Methoden:**

PmCI und normalhörende Proband*innen (NH) absolvierten den Oldenburger Satztest (OLSA) in Deutsch und in der Fremdsprache Englisch. Erfasst wurden der *SNR*_*50*_ (Signal-Rausch-Verhältnis) und die subjektive mentale Anstrengung, gemessen mittels der Einschätzungsskala Rating Scale Mental Effort (RSME). Außerdem wurde die subjektive Sprachkompetenz in der Fremdsprache Englisch mithilfe des Gemeinsamen Europäischen Referenzrahmens für Sprachen (GER) erhoben.

**Ergebnisse:**

Insgesamt wurden 28 Personen mit Deutsch als Erstsprache und Englisch als Fremdsprache (14 PmCI, 14 NH) einbezogen. Für die PmCI war der OLSA in Deutsch signifikant besser verständlich als in Englisch (*p* = 0,010), während sich bei den NH kein signifikanter Unterschied zwischen den Sprachbedingungen zeigte. Das Anstrengungsempfinden war sowohl bei PmCI (*p* = 0,003) als auch bei NH (*p* = 0,003) bei der Durchführung des OLSA in Englisch signifikant höher als bei der Durchführung des OLSA in Deutsch. Ein Zusammenhang zwischen subjektiv eingeschätzter Sprachkompetenz in Englisch und empfundener Anstrengung konnte in keiner Gruppe festgestellt werden.

**Schlussfolgerung:**

Die signifikant schlechtere Performanz von PmCI im OLSA im Störschall unter fremdsprachlichen Bedingungen verdeutlicht, dass mehrsprachige PmCI im Störschall stärker beeinträchtigt sind. Die zusätzlich reduzierte Automatisierung sprachlicher Verarbeitung sowie eine eingeschränkte Nutzung von Top-down-Hörstrategien, also der Nutzung von Vorwissen, Kontext und Erwartungen zum Schließen von Lücken im akustischen Signal, erschweren das Verstehen bei Hintergrundgeräuschen, was zu höherer Anstrengung und vermehrten Hörverständnislücken führen kann. Diese Effekte scheinen bei mehrsprachigen Personen besonders ausgeprägt. Dies verdeutlicht die Relevanz einer individualisierten, sprachlich und kulturell sensiblen Versorgung von PmCI in der klinischen Routine.

## Abstract (en)

**Background and objective:**

Speech comprehension in a foreign language under noise conditions presents an increased cognitive demand. For multilingual patients with cochlear implants (PwCI), this poses a particular challenge, as audiological routine diagnostics are typically conducted in the language of the clinical environment. This study investigates speech understanding in noise as well as the subjectively perceived listening effort in PwCI compared to normal-hearing (NH) individuals under both native and nonnative language conditions.

**Materials and methods:**

PwCI and NH completed the Oldenburg Sentence Test (OLSA) in both German and English. The SNR_50_ and the subjectively perceived mental effort, measured using the Rating Scale Mental Effort (RSME), were assessed. In addition, the subjective language competence in English as a foreign language was collected using the Common European Framework of Reference for Languages (CEFR).

**Results:**

A total of 28 individuals with German as a first language and English as a foreign language (14 PwCI, 14 NH) were included. Among PwCI, the German version of the OLSA was significantly more intelligible than the English version (*p* = 0.010), whereas no significant difference was found for NH between language conditions. Listening effort was significantly higher during the English version of the OLSA in both PwCI (*p* = 0.003) and NH (*p* = 0.003). No correlation was found between self-assessed English language proficiency and perceived effort in either group.

**Conclusion:**

The significantly reduced performance of PwCI in their foreign language under noise conditions reflects the established finding that multilingual individuals experience greater difficulty understanding speech in noise. The additionally reduced automatization of linguistic processing as well as a limited use of top-down listening strategies, that is the use of prior knowledge, context and expectations to fill gaps in the acoustic signal, make understanding in the presence of background noise more difficult, which can lead to increased listening effort and more frequent comprehension gaps. These effects appear to be particularly pronounced in multilingual individuals. These results highlight the importance of individualized, linguistically and culturally sensitive approaches in the clinical management of PwCI.

Angesichts globaler Migrationsbewegungen und sprachlicher Diversität begegnen Fachkräfte im klinischen Alltag immer häufiger Patient*innen, deren dominante Alltagssprache nicht der Umgebungssprache entspricht. Audiometrische Routinediagnostik erfolgt jedoch zumeist in der jeweiligen Landessprache, ohne die Mehrsprachigkeit der Patient*innen systematisch zu erfassen. Dies birgt das Risiko einer verzerrten Einschätzung der tatsächlichen Sprachverständnisleistung – mit potenziellen Folgen für Diagnostik, Beratung und Therapie. Vor diesem Hintergrund gewinnt die Berücksichtigung sprachlicher Faktoren im Rahmen der Patient*innenversorgung zunehmend an klinischer Relevanz.

## Einleitung

Weltweit betrachtet ist Mehrsprachigkeit die Normalsituation. Im Rahmen der Globalisierung und bedingt durch Fluchtbewegungen ist ein Großteil der Weltbevölkerung in der Lage, in mehr als einer Sprache zu kommunizieren, oder lebt in einem Land, in dem eine andere Umgebungssprache gesprochen wird als die eigene Erstsprache. In Deutschland leben derzeit etwa 21,1 Mio. Menschen mit Migrations- und einem damit oft einhergehenden mehrsprachigen Hintergrund [6]. Dies trifft auf etwas mehr als ein Viertel der Patient*innenklientel in Deutschland zu. Die in diesem Zusammenhang am häufigsten gesprochenen Sprachen sind Türkisch (15 %), Russisch (13 %), Arabisch (10 %) und Polnisch (7 %) [6].

Im Rahmen der Cochleaimplantat(CI)-Versorgung stehen Behandelnde und Patient*innen mit CI (PmCI) vor diversen Herausforderungen. Eine große Hürde im Behandlungsprozess stellt die Sprachbarriere zwischen den Behandelnden und den PmCI mit Migrationshintergrund (MH) dar [7, 9, 15]. Im Rahmen der Hördiagnostik sowie der Befund- und Beratungsgespräche werden kommunikative Schwierigkeiten deutlich. Der CI-Versorgungsprozess sowie die audiometrische Diagnostik sind für PmCI mit MH herausfordernd, weil die Sprachkompetenz und die sprachliche Performanz in der Umgebungssprache individuell variieren. Die Sprachkompetenz in der Umgebungssprache hat dabei einen großen Einfluss auf das Sprachverstehen. Studien zeigen, dass für die Beurteilung der sprachlichen Performanz mehrsprachiger Personen das Spracherwerbsalter und der Kenntnisstand der Sprachen von Bedeutung sind [8, 10]. Für Menschen mit mehrsprachigem Hintergrund führt eine Verschlechterung des Signal-Rausch-Verhältnisses (SNR) außerdem zu einem verschlechterten Sprachverstehen. Das Sprachverstehen im Störschall ist für diese Patient*innengruppe erschwert, wenn das Gehörte nicht in ihrer Erstsprache präsentiert wird, da das Sprachverstehen in einer Fremd- oder Zweitsprache unter Hinzugabe eines Störgeräusches abnimmt [2, 4]. Das Störgeräusch beeinflusst das Sprachverstehen in beiden Sprachen, in Abhängigkeit von der jeweiligen Sprachkompetenz [5]. Die Abnahme des Sprachverstehens aufgrund des Störgeräuschs ist größer, je geringer die Sprachkenntnisse sind [2, 5, 13]. Ein früher, simultaner Spracherwerb scheint das Sprachverstehen in geräuschvollen Hörsituationen für einen möglichst gleichwertigen Hörerfolg in beiden Sprachen allerdings zu begünstigen [14].

Der Einfluss, den Störschall auf mehrsprachige PmCI mit MH im Rahmen der Diagnostik und CI-Nachsorge hat, bleibt unklar. Die audiometrische Routinediagnostik ist oftmals in der jeweiligen Landessprache normiert und berücksichtigt mehrsprachige Sprachprofile aktuell kaum. Dies kann zu potenziellen Verzerrungen der Ergebnisse führen, da die sprachlichen und auditiven Kompetenzen von mehrsprachigen PmCI mit MH, deren Erstsprache nicht die Testsprache ist, kaum abgebildet werden können. Die Diagnostikergebnisse in der Fremdsprache sind demnach nicht gleichzusetzen mit Ergebnissen, die in der Erstsprache ermittelt wurden. Insbesondere im Kontext der CI-Versorgung kann diese Limitation die Erhebung relevanter Befunde und die anschließende Beratung sowie therapeutische Maßnahmen beeinflussen.

Ziel der Studie war deshalb die experimentelle Untersuchung des Sprachverstehens im Störschall von PmCI im Vergleich zu einer normalhörenden Kontrollgruppe (NH) im erstsprachlichen und im fremdsprachlichen Setting des Oldenburger Satztests (OLSA) [16]. Zudem wurde geprüft, ob sich das subjektive Anstrengungsempfinden von PmCI und NH beim Sprachverstehen im Störschall im erst- und fremdsprachlichen Setting unterscheidet.

## Material und Methoden

Die Studie wurde in Übereinstimmung mit der Deklaration von Helsinki durchgeführt. Ein positives Ethikvotum wurde von der Ethikkommission der medizinischen Fakultät der Heinrich-Heine-Universität Düsseldorf (2023-2429) erteilt. Alle Proband*innen wurden umfassend über die Ziele der Studie sowie das Studienprotokoll informiert. Die Studienteilnahme erfolgte freiwillig und ohne Vergütung. Eine Aufnahme in die Studie erfolgte ausschließlich nach schriftlicher Einwilligungserklärung. Ein Eintrag in das DRKS-Studienregister erfolgte unter der Nummer 00033927.

### Proband*innen

In die Studiengruppe wurden PmCI einbezogen, die sich zum Testzeitpunkt in der ambulanten CI-Nachsorge befanden, der Studienteilnahme zustimmten und folgende Einschlusskriterien erfüllten: Sie trugen mindestens ein CI oder waren bilateral mit CI versorgt, sie gaben an, Englisch als Fremdsprache zu beherrschen, die CI-Erstanpassung lagt mindestens 6 Monate zurück, sie nutzten ausschließlich lautsprachliche Kommunikation und gaben an, zum Testzeitpunkt keine Diagnosen kognitiver Beeinträchtigung zu haben.

Auf die Einbeziehung mehrsprachiger Proband*innen wurde bewusst verzichtet, da interindividuelle Unterschiede hinsichtlich Sprachdominanz, Erwerbszeitpunkt sowie sprachlicher Exposition eine standardisierte Testdurchführung und Vergleichbarkeit der Ergebnisse erschwert hätten. Stattdessen wurde zur Abbildung eines fremdsprachlichen Hörsettings der OLSA in englischer Sprache eingesetzt. Dies ermöglichte eine kontrollierte Untersuchung des Sprachverstehens im Störschall sowie des Anstrengungsempfindens unter reproduzierbaren Bedingungen.

Die Kontrollgruppe bestand aus NH, die ebenfalls angaben, dass sie Englisch als Fremdsprache beherrschen. Die Gruppe der NH nutzte ausschließlich lautsprachliche Kommunikation und gab an, zum Testzeitpunkt keine kognitive Beeinträchtigung zu haben.

### Design und Material

Die Studie wurde als experimentelle prospektive Studie konzipiert, in der beide Gruppen (PmCI und NH) sowohl den OLSA in Englisch als auch in Deutsch im Störschall absolvierten. Innerhalb der Gruppen fand die Durchführung des OLSA im Cross-over-Design statt, um einen beeinflussenden Effekt der jeweiligen Testung zu vermeiden. Für den OLSA lagen Normdaten in Englisch und Deutsch vor. Der OLSA wurde im geschlossenen Setting und adaptiv durchgeführt. Die Darbietung von Sprache und Störschall erfolgte in einer Hörkabine im freien Schallfeld aus frontaler Richtung (S_0_°/N_0_°) aus einem Tischlautsprecher in 1 m Entfernung. PmCI mit bilateraler CI-Versorgung wurden mit beiden CI-Systemen gemessen. Bimodal versorgte PmCI wurden mit einem CI und einem unversorgten Gegenohr (hochgradig Schwerhörig, an Taubheit grenzend Schwerhörig oder Gehörlos) gemessen (Tab. 1).

**Tab. 1:** (Gesundheitsbezogene) Informationen zur Gruppe der Patient*innen mit Cochleaimplantat (PmCI) und der Kontrollgruppe (NH)

	Gruppe	
	PmCI	NH
	*n* = 14	*n* = 14
Geschlecht ♀|♂ (%)	64,29|35,71	71,43|28,57
Alter zum Testzeitpunkt (Jahre) (M ± SD)	48,14 ± 17,42	40,71 ± 11,30
CI-Versorgungsdauer (Monate) (M ± SD)	72,00 ± 76,87	–
*CI-System (%)*	**–**	
Cochlear	50,00	
MED-EL	35,71	
Advanced Bionics	14,29	
*Versorgungsart (%)*	**–**	
Bilaterale CI-Versorgung	50,00	
Bimodale-Versorgung*	50,00	
*Audiometrische Daten*	**–**	
4PTA (M ± SD)	28,21 ± 5,74	3,75 ± 3,10
Sprachverstehen*** 65 dB (M ± SD)	87,50 ± 5,26	–
Sprachverstehen*** 80 dB (M ± SD)	93,93 ± 6,32	
*NCIQ (Gesamtwert in %)*	71,30 ± 15,47	
Elementare Geräuschwahrnehmung (%)	76,00 ± 12,13	
Fortgeschrittene Geräuschwahrnehmung (%)	71,39 ± 14,20	

*Unilaterales CI-System + Hörgerät auf dem Gegenohr (unversorgtes Gegenohr wurde hochgradig Schwerhörig, an Taubheit grenzend schwerhörig oder gehörlos gemessen), **bei bilateral CI-versorgten Proband*innen wurde das zuletzt implantierte CI berücksichtigt, ***die Messung erfolgte mit dem Freiburger Einsilbertest in Ruhe; bei bilateral CI-versorgten Proband*innen wurde das zuletzt implantierte CI berücksichtigt

Das Sprachmaterial des OLSA setzt sich aus Testlisten mit je 20 Sätzen zusammen. Die in dieser Studie durchgeführte Messreihe beinhaltete eine Trainingsliste und eine danach folgende Testliste. Die Testsätze zeigen dabei folgenden Aufbau: Name-Verb-Zahlwort-Adjektiv-Objekt. Für jede der fünf Satzpositionen existieren 10 Wortalternativen, die miteinander kombiniert werden. Durch den sich daraus zumeist ergebenden fehlenden Sinngehalt der Sätze kann ein Wiedererkennungseffekt des gesamten Satzes anhand eines einzelnen Wortteils ausgeschlossen werden. Bei der Ermittlung der Sprachverständnisleistung wurde den Proband*innen zeitgleich zu den Sätzen ein sprachsimulierendes Rauschen, das Bestandteil des Testmaterials OLSA ist, als Störschall dargeboten. Das sprachsimulierende Rauschen als Störschall wird im OLSA durch die Überlagerung des Sprachmaterials gebildet. Dies soll eine Alltagssituation realitätsnah nachbilden. Der Pegel des Störgeräuschs blieb dabei konstant, wobei ein Rauschpegel von 65 dB verwendet wurde. Die Sprachverständlichkeitsschwelle im Störgeräusch wurde mittels einer adaptiven Steuerung ermittelt. Die Sprachverständlichkeitsschwelle entspricht dem Signal-Rausch-Abstand, bei dem die Testperson 50 % der dargebotenen Wörter richtig verstehen kann (*SNR*_*50*_), und wird als Ergebnis der Messung im Störgeräusch angegeben. Der Pegel der Sprache wurde entsprechend der Antwort der Proband*innen durch das OLSA-System verändert, um die Sprachverständlichkeitsschwelle möglichst effizient zu bestimmen.

Der Hörverlust wurde als Mittelwert über die vier Oktavfrequenzen 0,5, 1, 2 und 4 kHz berechnet und als Vier-Frequenz-Mittelwert (4PTA, dB HL) angegeben, wobei die Messung im Freifeld mit pulsierendem Schmalbandrauschen erfolgte. Die Sprachverständlichkeit im Freifeld wurde für das CI-versorgte Ohr bei 65 und 80 dB Schalldruckpegel mithilfe des Freiburger Einsilbertests unter Ruhebedingungen erfasst. Bei bilateral versorgten PmCI wurde das zuletzt implantierte CI gemessen.

Das Anstrengungsempfinden wurde mittels der Einschätzungsskala Rating Scale Mental Effort (RSME) erhoben [18]. Die RSME ist eine etablierte eindimensionale, visuelle Analogskala von 0 (keine Anstrengung) bis 150 (unerträgliche Anstrengung) zur subjektiven Einschätzung kognitiver Beanspruchung während der Bearbeitung kognitiv fordernder Aufgaben. Zur Orientierung sind verbale Marker entlang der Skala platziert. Die RSME erlaubt eine sensitive und differenzierte Erfassung der empfundenen kognitiven Anstrengung und kann zur Quantifizierung subjektiver Belastung in verschiedenen experimentellen Bedingungen eingesetzt werden.

Alle Proband*innen schätzten vor Beginn des Experiments ihre Englischkenntnisse strukturiert selbst ein. Die Selbsteinschätzung erfolgte auf Basis des Gemeinsamen Europäischen Referenzrahmens für Sprachen (GER) [3]. Der GER ist ein international anerkannter Standard zur strukturierten Bewertung sprachlicher Kompetenzen und unterteilt diese in 6 Niveaustufen: A1, A2, B1, B2, C1 und C2 (Council of Europe, 2001). Diese Stufen reichen von einer elementaren (A1/A2) und selbstständigen Sprachverwendung (B1/B2) bis hin zu einer kompetenten Sprachverwendung (C1/C2) und ermöglichen eine systematische Erfassung der sprachlichen Fähigkeiten Hören, Lesen, an Gesprächen teilnehmen, zusammenhängendes Sprechen und Schreiben. Jeder sprachlichen Fähigkeit ordneten die Proband*innen eine der 6 Niveaustufen zu. Anschließend wurde aus den Ergebnissen ein Mittelwert gebildet.

Demographische Daten wurden zusätzlich aus der medizinischen Patient*innenakte erhoben.

Die Auswertungen erfolgten mit dem Statistikprogramm IBM SPSS Statistics (für Windows), Version 29.0.1 sowie mit Microsoft Excel. Als Signifikanzniveau wurde *p* < 0,05 festgelegt. Die Effektstärken wurden nach den Konventionen von Cohen (1988) interpretiert: Werte *r* < 0,3 gelten als kleiner Effekt, Werte *r* ≤ 0,3 – < 0,5 gelten als mittlerer Effekt und Werte *r* ≥ 0,5 gelten als großer Effekt.

## Ergebnisse

### Proband*innen

14 PmCI und 14 NH konnten in die Studie einbezogen werden (*n* = 28). Die PmCI hatten zum Testzeitpunkt ein Durchschnittsalter von 48,14 Jahren (SD = 17,42) und waren im Durchschnitt 72 Monate (SD = 76,80) mit einem oder zwei CI versorgt (Tab. 1). Die PmCI wiesen auf dem CI-versorgten Ohr einen 4PTA von 28,21 dB HL (SD = 5,74) auf. Bei bilateral versorgten PmCI wurden die Ergebnisse des zuletzt implantierten CI berücksichtigt. Die Auswertung des Freiburger Einsilbertests im Freifeld in Ruhe ergab bei 65 dB SPL ein Sprachverstehen von 87,50 % und bei 80 dB SPL von 93,93 %. Alle PmCI erzielten ein sehr gutes Sprachverstehen [11]. Für die PmCI lagen zudem aktuelle Werte des Nijmegen Cochlear Implant Fragebogens (NCIQ) vor, der im Rahmen der leitliniengerechten Versorgung in CI-versorgenden Einrichtungen erhoben wird. Diese Daten sind nicht Teil des eigentlichen Studiendesigns, werden hier jedoch beschreibend genutzt, um die Studiengruppe möglichst ganzheitlich zu charakterisieren und aufzuzeigen, wie die PmCI ihre Hör- und Klangqualität subjektiv einschätzen. Die Auswertung des NCIQ-Gesamtwertes sowie der Subskalen zur elementaren und fortgeschrittenen Geräuschwahrnehmung der PmCI ergaben Werte von im Durchschnitt über 70 % (Tab. 1). Diese lassen Rückschlüsse auf eine subjektiv gute bis sehr gut Hör- und Klangqualität der PmCI zu.

Die Analyse des Bildungsniveaus der PmCI zeigte, dass jeweils 42,86 % über einen Haupt- oder Realschulabschluss bzw. die Fachhochschulreife oder das Abitur verfügten. Ein universitärer Bildungsabschluss lag bei 14,29 % der PmCI vor.

Die Kontrollgruppe umfasste 14 NH, von denen 10 weiblich und 4 männlich waren. Diese waren zum Testzeitpunkt im Durchschnitt 41 Jahre alt (SD = 11,30). In der Kontrollgruppe NH verfügten 14,29 % über einen Haupt- oder Realschulabschluss, weitere 14,29 % über die Fachhochschulreife oder Abitur. Ein Anteil von 71,43 % hatte einen universitären Bildungsabschluss.

### OLSA

Der OLSA wurde in Deutsch und in Englisch im Störgeräusch durchgeführt. Den Autor*innen ist bekannt, dass PmCI im Störschall nicht die Performanz und Resultate zeigen können wie NH. Dies wird durch zahlreiche Studien bestätigt und ist auf die bislang noch begrenzte Möglichkeit zurückzuführen, auch diffizile Höranteile, die über das bloße Verstehen hinausgehen, apparativ durch Hörimplantate nachzubilden [12]. Dennoch wurde der Vollständigkeit halber der Unterschied berechnet (Tab. 2).

**Tab. 2:** Ergebnisse der OLSA-Messung

Gruppe	*n*	*Mdn*	*M*	*SD*	*Min*	*Max*
PmCI OLSA Deutsch	14	−1,10	−1,46	2,43	2,60	−6,10
NH OLSA Deutsch	14	−8,15	−8,22	0,78	−6,90	−9,50
PmCI OLSA Englisch	12	0,60	1,98	4,37	0,60	−3,20
NH OLSA Englisch	14	−7,70	−7,11	2,21	−0,80	−9,10

*OLSA* Oldenburger Satztest, *PmCI* Patient*innen mit Cochleaimplantat, *NH* normalhörende Kontrollgruppe

Ein Mann-Whitney-U-Test wurde berechnet, um zu überprüfen, ob die PmCI im Vergleich zur NH-Gruppe im Median abweichende Ergebnisse im OLSA in der deutschen Version erreichen. Es konnte ein signifikanter Unterschied zwischen den Gruppen nachgewiesen werden (*Z* = −4,505, *p* < 0,001, *n* = 14).

Die Effektstärke betrug *r* = 1,204, was auf einen großen Effekt hindeutet. Der mittlere Rangwert für den *SNR*_*50*_ der PmCI lag bei −1,10 dB, während er für die NH-Kontrollgruppe bei −8,15 dB lag.

Ein Mann-Whitney-U-Test wurde ebenfalls berechnet, um zu überprüfen, ob die PmCI im Vergleich zur NH-Gruppe im Median abweichende Ergebnisse im OLSA in der englischen Version erreichen. Auch in dieser Berechnung konnte ein signifikanter Unterschied für die beiden Gruppen nachgewiesen werden (*Z* = −4,221, *p* < 0,001, *n* = 12). Die Effektstärke betrug *r* = 1,128, was ebenfalls auf einen starken Effekt hindeutet. Der *SNR*_*50*_ betrug für die Gruppe der PmCI im Median 0,60 dB, während der mediane *SNR*_*50*_ der NH −7,70 dB betrug. In die Berechnung konnten *n* = 12 PmCI und *n* = 14 NH einbezogen werden. Zwei PmCI haben die Durchführung des OLSA in Englisch vor Beendigung der Messung aufgrund von zu hoher Anstrengung abgebrochen.

Mittels des Wilcoxon-Tests für verbundene Stichproben wurde zudem überprüft, ob PmCI im OLSA in Englisch und im OLSA in Deutsch unterschiedlich performen. Für den OLSA in Englisch betrug der *SNR*_*50*_ im Median 0,60 dB. Der OLSA in Deutsch war mit einem medianen *SNR*_50_ von −1,10 dB für die PmCI damit besser verständlich (*Z* = −2,589, *p* = 0,010, *n* = 26). Die Effektstärke liegt bei *r* = 0,507 und entspricht einem starken Effekt. Die NH erzielten im OLSA in Deutsch hingegen keinen signifikant abweichenden *SNR*_*50*_ (Mdn = −8,15 dB) als im OLSA in Englisch (Mdn = −7,70 dB; *Z* = 1,695, *p* = 0,090, *n* = 28). Die Effektstärke liegt bei *r* = 0,320 und entspricht einem moderaten Effekt.

### Anstrengungsempfinden

**Abb. 1:**
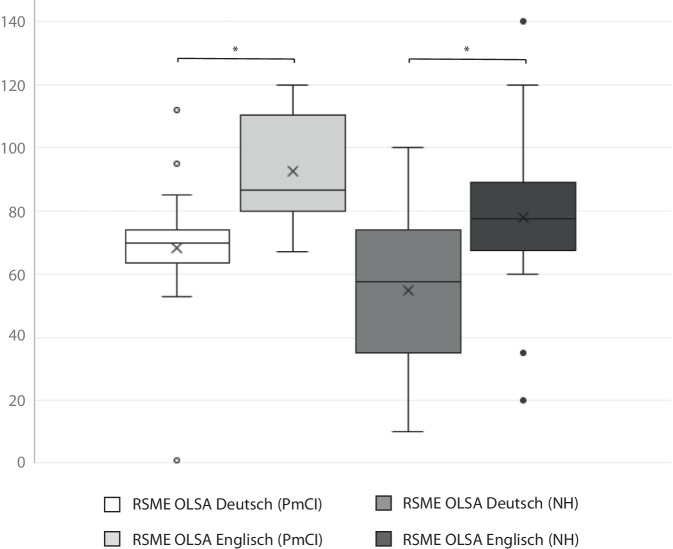
Ergebnisse zum Anstrengungsempfinden, erhoben mittels RSME. (Anmerkung. Die *Ordinatenachse* zeigt die zu erreichenden RSME-Werte (0 = keine Anstrengung bis 150 = unerträgliche Anstrengung). Auf der *Abszissenachse* sind die Ergebnisse der OLSA-Gruppenvergleiche der Proband*innen mit Cochleaimplantat (*PmCI*) und der Kontrollgruppe (*NH*) für den OLSA in Englisch und Deutsch dargestellt. Das x markiert den Mittelwert. Der *Asterisk* markiert die Signifikanzen)

Wie in Abb. 1 dargestellt, empfanden die PmCI bei der Durchführung des OLSA in Englisch signifikant höhere Anstrengung (Mdn = 86,50) als bei der Durchführung des OLSA in Deutsch (Mdn = 70,00; Wilcoxon-Test: *Z* = 2,934, *p* = 0,003, *n* = 28). Die Effektstärke liegt bei *r* = 0,554 und entspricht einem starken Effekt. Auch die NH-Kontrollgruppe empfindet bei der Durchführung des OLSA in Englisch signifikant höhere Anstrengung (Mdn = 77,50) als bei der Durchführung des OLSA in Deutsch (Mdn = 57,50; Wilcoxon-Test: Z = 2,950, *p* = 0,003, *n* = 28). Die Effektstärke liegt bei *r* = 0,577 und entspricht ebenfalls einem starken Effekt.

Zur Untersuchung des Zusammenhangs zwischen Hörverlust (angegeben als 4PTA in dB HL) und subjektiv empfundener mentaler Anstrengung, gemessen anhand der RSME, wurde eine partielle Korrelationsanalyse durchgeführt. Das Alter zum Testzeitpunkt wurde dabei kontrolliert. Für den OLSA in deutscher Sprache ergab sich kein statistisch signifikanter Zusammenhang zwischen dem Hörverlust und der subjektiven Anstrengung (*r*(11) = 0,380; *p* = 0,201; *n* = 14), was auf keinen bedeutsamen Zusammenhang zwischen den Variablen hinweist. Ebenso zeigte sich im englischen OLSA kein signifikanter Zusammenhang (*r*(11) = 0,070; *p* = 0,820; *n* = 14). Die Korrelationskoeffizienten von 0,380 (OLSA Deutsch) bzw. 0,070 (OLSA Englisch) deuten darauf hin, dass unter Kontrolle des Alters zum Testzeitpunkt ein zunehmender Hörverlust nicht mit einer höheren subjektiven mentalen Anstrengung assoziiert ist.

### GER

**Abb. 2:**
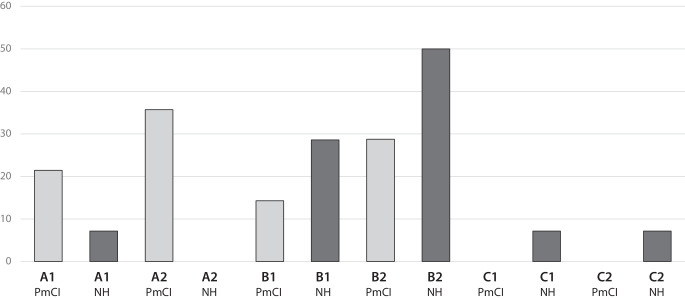
Einschätzung der Fremdsprachkompetenz in Englisch der Proband*innen mit Cochleaimplantat und der Kontrollgruppe im Vergleich. (Anmerkung. Die *Ordinatenachse* zeigt den prozentualen Anteil der Proband*innen. Auf der *Abszissenachse* ist die subjektive Fremdsprachkompetenz der Proband*innen mit Cochleaimplantat (*PmCI*) und der Kontrollgruppe (*NH*) in Englisch dargestellt. Die Einschätzung der Sprachkompetenz erfolgte durch die Proband*innen selbst anhand des Gemeinsamen Europäischen Referenzrahmens für Sprache (*GER*). Der GER teilt Sprachkompetenz in sechs Stufen ein, die drei Hauptniveaus zugeordnet sind: A (Elementare Sprachverwendung), B (Selbstständige Sprachverwendung) und C (Kompetente Sprachverwendung). Für die Stufen A2 (NH), C1 (PmCI) und C2 (PmCI) lagen keine Werte vor)

Die PmCI und die NH schätzten vor Beginn des Experiments ihre sprachlichen Fähigkeiten in ihrer Fremdsprache Englisch mittels des GER ein (Abb. 2). Etwa 57 % der PmCI schätzten ihre fremdsprachlichen Fähigkeiten in Englisch als elementar (A1/A2) ein. In der Gruppe der NH schätzte nur eine Person ihre sprachlichen Fähigkeiten in der Fremdsprache als elementar (A1) ein. Rund 43 % der PmCI schätzten ihre Sprachverwendung in der Fremdsprache als selbstständig (B1/B2) ein, während es bei den NH etwa 79 % waren. Nur eine Person in der Gruppe der NH schätzte ihre sprachlichen Fähigkeiten in Englisch als kompetent (C1) ein. Insgesamt schätzte die Gruppe der NH ihre sprachlichen Fähigkeiten in ihrer Fremdsprache Englisch subjektiv besser ein als die PmCI, was durch den insgesamt höheren Bildungsgrad und die damit zumeist längere schulische Kontaktzeit mit der Fremdsprache der NH begründet werden kann (Tab. 1).

Zur Untersuchung des Zusammenhangs zwischen der subjektiven Sprachkompetenz (erhoben mittels GER) und der subjektiv empfundenen mentalen Anstrengung bei der Durchführung des OLSA in englischer Sprache wurde für die PmCI eine partielle Korrelationsanalyse durchgeführt. Der Bildungsgrad der Proband*innen wurde dabei als Kontrollvariable berücksichtigt. Die Analyse ergab keinen statistisch signifikanten Zusammenhang zwischen dem angegebenen Sprachniveau der PmCI in ihrer Fremdsprache Englisch und dem subjektiven Anstrengungsempfinden bei der Durchführung des OLSA in Englisch (*r*(11) = −0,032; *p* = 0,917; *n* = 14), was auf das Fehlen einer Assoziation zwischen beiden Variablen hinweist. Der Korrelationskoeffizient von −0,032 deutet darauf hin, dass unter Kontrolle des Bildungsniveaus eine höhere subjektive Sprachkompetenz nicht mit einer geringeren subjektiven mentalen Anstrengung assoziiert ist.

Im Rahmen einer partiellen Korrelationsanalyse wurde innerhalb der NH der Zusammenhang zwischen der selbst eingeschätzten Sprachkompetenz (gemessen anhand des GER) und der subjektiv wahrgenommenen mentalen Anstrengung während der Durchführung des OLSA in englischer Sprache untersucht. Als Kovariate wurde der formale Bildungsabschluss berücksichtigt. Die Analyse ergab keinen statistisch signifikanten Zusammenhang zwischen Sprachkompetenz und subjektiver mentaler Anstrengung (*r*(11) = −0,275; *p* = 0,364; *n* = 14). Dieser Befund deutet darauf hin, dass unter Kontrolle des Bildungsniveaus ebenfalls keine systematische Assoziation zwischen dem Grad der Sprachkompetenz und dem Ausmaß der empfundenen Belastung besteht. Der Korrelationskoeffizient von r = −0,275 lässt demnach keine Evidenz für einen Zusammenhang erkennen, der auf eine verminderte mentale Anstrengung bei höherer Sprachkompetenz im Rahmen des Sprachverstehens in der Fremdsprache schließen ließe.

Zur Analyse des Zusammenhangs zwischen der Sprachkompetenz und der Leistung im OLSA in englischer Sprache wurde für die Gruppe der PmCI eine Spearman-Rho-Korrelationsanalyse durchgeführt. Die Auswertung ergab keinen statistisch signifikanten Zusammenhang zwischen den betrachteten Variablen (ρ(12) = −0,309; *p* = 0,328).

Darüber hinaus wurde auch für die Kontrollgruppe eine Spearman-Rho-Korrelationsanalyse durchgeführt, um den Zusammenhang zwischen der Sprachkompetenz und der Performanz im englischen OLSA zu untersuchen. In dieser Gruppe zeigte sich ein starker und statistisch signifikanter negativer Zusammenhang zwischen beiden Variablen (ρ(14) = −0,553; *p* = 0,040).

## Diskussion

Die vorliegende Studie ermittelte die Höranstrengung und Performanz von PmCI im erst- und fremdsprachlichen Setting. Die Ergebnisse tragen wesentlich zur aktuellen Diskussion über die Herausforderungen mehrsprachiger PmCI bei, insbesondere im Kontext der zunehmenden Globalisierung und Migration. Wirtschaftliche, soziale und politische Konflikte sowie Veränderungen der Umwelt bedingen die weltweite Zunahme humaner Migration [17]. Einwanderungsländer wie Deutschland oder Frankreich stehen vor der Herausforderung, mehrsprachige Menschen mit MH in das bestehende Gesundheitssystem zu integrieren. Gründe für auftretende Schwierigkeiten finden sich oft in der bestehenden Sprachbarriere, die vor allem in sprachaudiometrischen Untersuchungen zum Hindernis wird.

PmCI erzielten in dieser Studie signifikant schlechterer Ergebnisse im OLSA in Englisch im Störschall als in ihrer Erstsprache Deutsch. Ähnliche Ergebnisse zeigen bereits frühere Studien, die herausfanden, dass Menschen mit mehrsprachigem Hintergrund erhöhte Schwierigkeiten beim Hören im Störschall haben [2, 5]. Ebenso wird die Durchführung des OLSA in Englisch als anstrengender empfunden als die Durchführung des OLSA in der Erstsprache. PmCI zeigen in alltäglichen Hörsituationen eine erhöhte Höranstrengung gegenüber NH [1]. Das erhöhte Anstrengungsempfinden in der Fremdsprache gegenüber der Erstsprache kann demnach als zusätzlich gesteigerte Anstrengung interpretiert werden, da davon auszugehen ist, dass das Sprachverstehen in der Fremdsprache einen zusätzlichen Einfluss auf die Höranstrengung hat.

Bei den PmCI konnte in dieser Studie kein Zusammenhang zwischen dem Grad des Hörverlusts und dem Anstrengungsempfinden nachgewiesen werden, was möglicherweise auch mit der geringen Proband*innenanzahl begründet werden kann. Außerdem erzielten die PmCI, die an unserer Studie teilnahmen, sehr gute 4PTA-Werte. Hier wäre eine folgende Untersuchung mit PmCI, die weniger hohe 4PTA-Werte erzielen, sinnvoll.

Alle Proband*innen schätzten vor Beginn des Experiments ihre sprachlichen Fähigkeiten in ihrer Fremdsprache Englisch subjektiv ein. Die Ergebnisse zeigten ebenfalls keinen Einfluss des subjektiv eingeschätzten Sprachniveaus und dem Anstrengungsempfinden bei der Durchführung des englischen OLSA im Störschall. PmCI, die ihre sprachlichen Fähigkeiten als eher niedriger einschätzten, empfanden demnach keine verstärkte Anstrengung. Auch die Performanz im OLSA in Englisch scheint für die PmCI in keinem Zusammenhang mit dem selbst eingeschätzten Sprachniveau zu stehen. Allerdings muss auch hier die geringe Proband*innenanzahl beachtet werden, da sich in einer größeren Stichprobe gegebenenfalls Zusammenhänge zeigen könnten. Die Analyseergebnisse wären demnach in einer größeren Stichprobe zu überprüfen.

Klinische Studien wie die von Ellahham [7] und Timmins [15] heben hervor, dass Sprachbarrieren eine der größten Hürden in der Gesundheitsversorgung darstellen. Unsere Ergebnisse ergänzen diese Perspektive, indem sie zeigen, dass die sprachlichen Fähigkeiten der PmCI nicht nur die Kommunikation mit Behandelnden, sondern auch ihre Fähigkeit zur Verarbeitung von sprachlichen Informationen im diagnostischen Kontext beeinflussen.

### Limitationen

Trotz ihrer Relevanz weist die vorliegende Studie Einschränkungen auf, die bei der Interpretation der Ergebnisse berücksichtigt werden sollten. Die Stichprobengröße könnte als limitierend angesehen werden, insbesondere in Bezug auf die Homogenität der PmCI. Die PmCI, die an unserer Studie teilnahmen, zeigten sehr gute 4PTA-Werte und schätzen ihre elementare und fortgeschrittene Geräuschwahrnehmung im NCIQ als eher hoch ein. Auch die Ergebnisse im OLSA in Deutsch im Störschall deuten auf PmCI hin, die mit den CI eine gute Hörperformanz erreichen. Ferner wurden die Studie mit deutschsprachigen PmCI durchgeführt, die Englisch als Fremdsprache beherrschen. Dieses Vorgehen wurde ausgewählt, weil sich im klinisch-praktischen sowie dem forschungsorientierten Setting ein eingeschränkter Zugriff auf Routineaudiometrieverfahren in anderen Sprachen zeigt, was eine adäquate Diagnostik mehrsprachiger PmCI oder von PmCI mit MH erschwert. In monolingual geprägten Ländern wie Deutschland können die begrenzten diagnostischen Ressourcen nur schwer die verschiedenen sprachlichen Hintergründe und Migrationserfahrungen der PmCI adressieren. Zudem basieren die Ergebnisse auf Testungen, die unter Laborbedingungen durchgeführt wurden. Es bleibt unklar, inwieweit die Ergebnisse auf reale Kommunikationssituationen übertragbar sind, in denen zusätzliche Variablen wie nonverbale Kommunikation eine Rolle spielen.

## Fazit für die Praxis

Die vorliegende Arbeit bietet wichtige Impulse für die Forschung und Praxis zu mehrsprachigen PmCI und zu PmCI und MH und hebt die Bedeutung einer individualisierten, sprachlich und kulturell sensiblen Versorgung von PmCI hervor. Durch die Verknüpfung linguistischer und audiologischer Perspektiven bietet die Studie ein multidimensionales Verständnis der Problematik, dass Mehrsprachigkeit in der Routineaudiometrie bisher kaum bis gar nicht beachtet wird. Es scheint sinnvoll, standardisierte klinische Prozesse dahingehend zu überprüfen und bei Bedarf zu überarbeiten. Zukünftige Forschung sollte darüber hinaus den Einfluss von kulturellen und psychosozialen Faktoren auf die Hörwahrnehmung weiter untersuchen. Zusammenfassend leisten die Ergebnisse dieser Studie einen Beitrag zum Verständnis der komplexen Interaktion von Sprachkompetenz, kognitiver Belastung und audiologischer Performanz. Sie bestätigen nicht nur bestehende Forschungsergebnisse, sondern erweitern diese durch die spezifische Betrachtung von PmCI im mehrsprachigen Kontexten.

## Data Availability

Die im Rahmen dieser Studie generierten und analysierten Daten können auf Anfrage bei der korrespondierenden Autorin/dem korrespondierenden Autor eingesehen werden. Eine öffentliche Bereitstellung der vollständigen Datensätze ist derzeit nicht vorgesehen.
